# Characterization of sucrose binding protein as a seed-specific promoter in transgenic tobacco *Nicotiana tabacum* L.

**DOI:** 10.1371/journal.pone.0268036

**Published:** 2022-06-03

**Authors:** Nasibeh Chenarani, Abbasali Emamjomeh, Hassan Rahnama, Katayoun Zamani, Mahmoud Solouki

**Affiliations:** 1 Department of Plant Breeding and Biotechnology (PBB), Faculty of Agriculture, University of Zabol, Zabol, Iran; 2 Department of Bioinformatics, Laboratory of Computational Biotechnology and Bioinformatics (CBB Lab), University of Zabol, Zabol, Iran; 3 Department of Genetic Engineering & Biosafety, Agricultural Biotechnology Research Institute of Iran (ABRII), Agricultural Research Education and Extension Organization (AREEO), Karaj, Iran; McGill University, CANADA

## Abstract

Seed-specific expression using appropriate promoters is a recommended strategy for the efficiently producing valuable metabolites in transgenic plants. In the present study, we investigated the sequence of sucrose binding protein (SBP) as a seed-specific promoter to find the *cis*-acting elements specific to gene expression in seeds. The 1860 bp SBP sequence was analyzed using Plant Care and PLACE databases to find *cis*-acting elements, which resulted in a finding of 22 *cis*-acting elements required for seed expression. In addition, we have discovered *cis*- acting elements that are indirectly involved in triacylglycerol synthesis (GATABOX, DOFCOREZM, CACGTGMOTIF). The seed specificity of SBP was analyzed by generating a stable transgenic tobacco plant harboring β-glucuronidase (GUS) reporter gene under the control of the SBP promoter. Histochemical analysis of these transgenic tobacco plants indicated decreasing GUS activity in the leaves during the vegetative stage. However, the mature seeds of transgenic plants showed GUS activity. Moreover, the SBP promoter function in the seed oil content was evaluated by the expression of *DGAT1*. The expression analysis of *DGAT1* in *SBP-DGAT1* transgenic tobacco seeds using quantitative real-time PCR revealed a 7.8-fold increase in *DGAT1* than in non-transgenic plants. Moreover, oil content increased up to 2.19 times more than in non-transgenic plants. And the oil content of the *SBP-DGAT1* transgenic tobacco leaves did not change compared to the control plant. Therefore, we suggested that the SBP promoter could be used as a seed-specific promoter for targeted expression of desired genes in the metabolite engineering of oilseed crops.

## Introduction

The specific promoters are crucial in plant genetic engineering projects. Spatiotemporal, constitutive, and inducible promoters are three types based on their expression patterns. Spatiotemporal promoters, used for genetic engineering, lead to specific expression in targeted tissues or organs [1]. β‐conglycinin in soybean [2], *α*-globulin in cotton [3], γ-zein in maize [4], Glutenin in wheat [5], and *VvβVPE* in Vitis [6] were reported as seed-specific promoters in plants.

*Cis*-acting elements, are located upstream of the start coding site, regulate gene expression at the promoter level [7] and lead to specific expression in the seed [8–11]. It has been observed that *cis*-acting elements could enhance gene expression in a tissue-specific and temporally regulated manner during embryo development in plants [12].

The required carbon for fatty acids (FA) synthesis can be prepared via two routes: 1- glycolytic reactions, and 2- ribulose-1,5-bisphosphate carboxylase. Pyruvate and NADPH produced in light reactions, increase the carbon required to synthesis of fatty acids [13]. A common strategy to improve oil content is to enhance the metabolic flux of carbon to oil, which leads to increased triacylglycerol (TAG) synthesis within the developing seeds [14].

Another way is using the effect of sucrose carriers’ on plant development and carbon allocation controlling [15]. Therefore, increasing carbohydrate content and sucrose carriers may lead to higher TAG synthesis and oil content in plants.

Sucrose and hexose transporters (*i*.*e*., VfSUT1 and VfSTP1, respectively) as sucrose carriers, control carbohydrate availability during seed development of fava bean [16]. It was shown that, in developing seeds of *Brassica*. *napus* L., unloaded sucrose from the phloem was imported to the glycolytic route. Afterward, it was converted to intermediates like hexose-phosphates, phosphoenolpyruvate, and pyruvate that was transferred subsequently to plastid to be used in fatty acid synthesis [17].

Sucrose binding proteins (SBPs) were reported in the plasma membrane of various tissues in the ripe vegetables of soybean, play an essential role in the quality and indirectly yield [18]. SBP was identified as a 62 KD membrane protein in soybean cotyledons involved in sucrose translocation [19]. Two homologous *SBP* genes (*Y11207* and *VFA292221*) were isolated from pea and *Vicia faba*, respectively. Conserved regions between the *SBP* gene and vicilin-like protein have been reported. Vicilin-like protein contributes to the storage proteins of the seeds [20, 21].

The *de novo* TAG production from Glycerol-3-Phosphate (G3P) and acyl CoA is done through the Kennedy pathway [22, 23]. This pathway is carried out by four enzymes, including glycerol-3-phosphate acyltransferase (GPAT), lysophosphatidic acid acyltransferase (LPAAT), phosphatidic acid phosphatase (PAP), and diacylglycerol acyl CoA acyltransferase (DGAT). Moreover, the only enzyme committed to TAG synthesis is DGAT [22].

*LPAAT* [24], *GPAT* [25], and *AtDGAT1* [13, 26] are the individual genes of the Kennedy pathway and; their overexpression leads to a significant increase in oil content. Among various *DGAT* genes in *DGAT* gene families, *DGAT1* plays a more critical role in TAG biosynthesis in Soybean and *Arabidopsis* than others [27, 28].

It was shown that DGAT1 is a necessary enzyme in the biosynthesis of seed TAG and seed oil accumulation consequently [22]. The effect of the *GmDGAT1A* on TAG production in soybean seeds has been reported [29]. The consequence of *GmDGAT1A* on changes in the oils and fatty acid levels in plants is well studied [13, 28, 30–32].

Recently it was shown that seed-specific promotors led to improve oil content [33]. Moreover, using seed-specific promoter “napin” in *Brassica carinata* increased the amount of docosadienoic acid (DDA) and docosatrienoic acid (DTA) in the seeds by 30% in total fatty acid [33]. In addition, overexpression of *DGAT1* and *GPD1* genes using two seed-specific promoters, i.e., oleosin and glycinin led to an increase of up to 13% in seed oil content in *Camelina sativa* [34]. Therefore, the characterization of seed-specific promoters is very critical to developing transgenic plants, especially in cereal and oilseed crops whose seeds are the main products. According to various applications of plant seed oil, studying the quantity and quality of FA and TAG in these seeds is necessary.

The present study aim, was to identify the *cis*-elements and seed-specific motif sequences in the SBP sequence promoter. Regarding the fact that a desirable seed-specific promoter should be active in seeds with no effect on other tissues, and due to the importance of SBP in the production of FA and TAG; the specificity of the SBP during vegetative and reproductive stages, and its effect on DGAT1 expression on the TAG pathway and oil content in transgenic tobacco plants was measured.

This could introduce SBP as a promoter to overexpression of enzymes to further improve seed oil content in commercial oilseed crops.

## Materials and methods

### In silico promoter analysis

Initially, the SBP sequence was retrieved in PlantPromDB [35] to find probable homology. Then, the probable regulatory *cis*-elements in the SBP sequence were searched and recognized in two online tools: PLANTCARE [36] and PLACE [37]. The described function of each element, which was predicted by PLACE, was investigated to find seed-specific elements and other related elements in the TAG pathway.

### Construction of plant expression vectors

**Fig 1:**
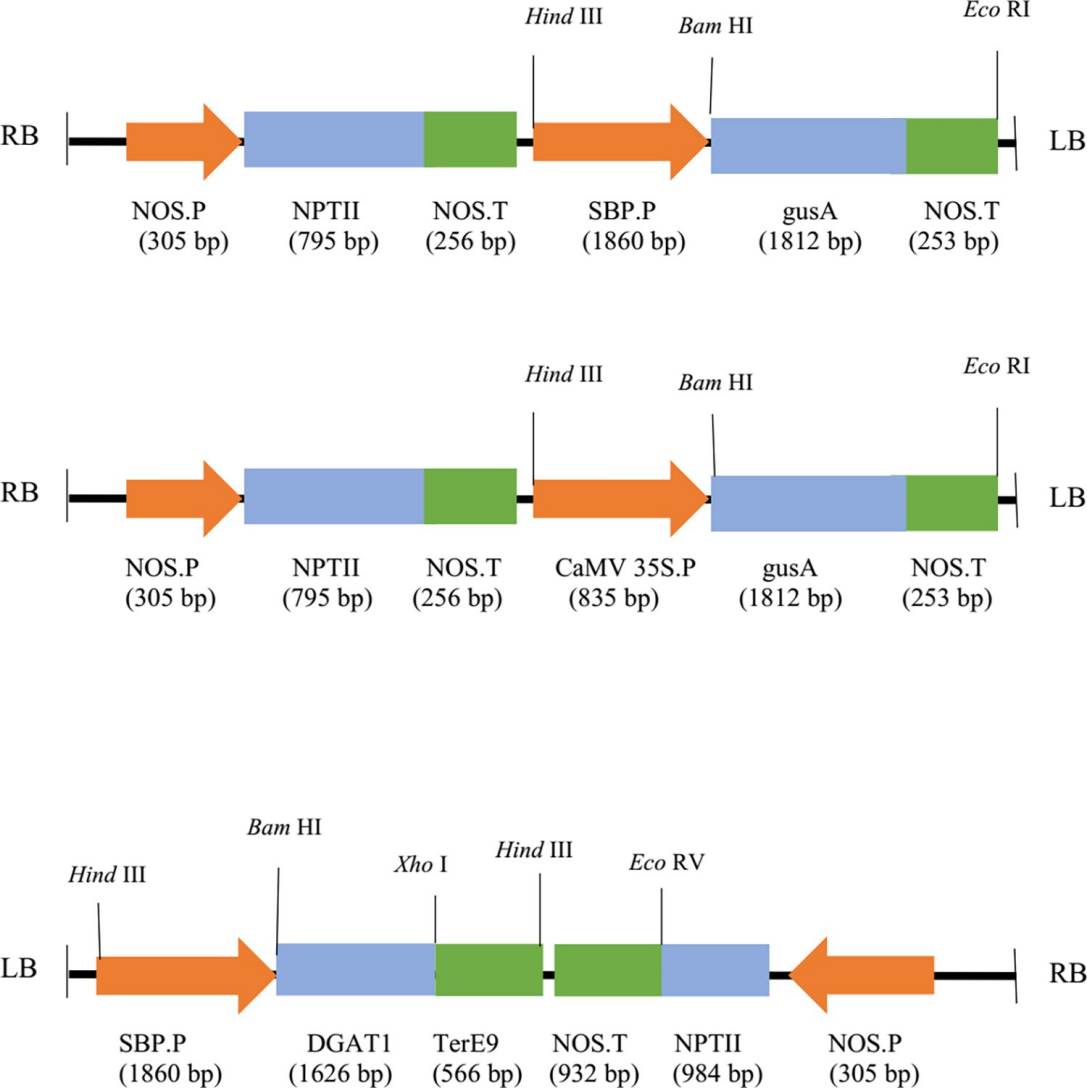
Construct maps: **a.** SBP- GUS **b.** 35S- GUS **c**. SBP- DGAT1; P. NOS: Nopaline synthase promoter; NPTII: Neomycin phosphotransferase gene; T. NOS: Nopaline synthase terminator; SBP.P: SBP promoter, gusA: β-glucuronidase gene; CaMV 35S.P: CaMV 35S promoter; DGAT1: Diacylglycerol acyl-CoA acyltransferase gene; TerE9: E9 terminator.

The *DGAT1* sequence (accession number KU744408.1) was used to construct the pSBP-DGAT1 following codon optimization based on the safflower sequence. SBP sequence with accession number AJ277287 from *Vicia faba* was used as a promoter sequence. Then, the constructs were designed by DNASTAR_lasergene software [38]. SBP (1860 bp) and DGAT1 (1626 bp) were synthesized by GENE ray company (China) in pGH vectors (i.e., pGH-SBP and pGH-DGAT1). CaMV35S promoter was replaced by SBP promoter in the upstream of GUS gene (Fig 1B) in pBI121 binary vector and named as pSBP-GUS (Fig 1A). The DGAT1 sequence was removed from pGH-DGAT1 vector by enzymatic digestion with *Avr*II and *Xho*I and cloned into the pGH-SBP vector and confirmed by enzyme digestion. Then this vector was digested with *Hind* III and sub-cloned into pBIN19, and the construct was named pSBP-DGAT1 (Fig 1C). pSBP-DGAT1, pSBP-GUS, and pBI121 vectors were transferred to *Agrobacterium tumefaciens* strain EHA105 by freeze-thawing [39] and were used to transform tobacco plants.

### The agrobacterium-mediated transformation of tobacco

The agrobacterium-mediated method was used to transform tobacco plants *Nicotiana tabacum* L., Var. Xanthi. Leaf discs from *in vitro* grown plants were inoculated for 15 min with an overnight culture of *A*. *tumefaciens* EHA105 containing pSBP-DGAT1 and SBP-GUS constructs [40]. After two-day co-cultivation in (Murashige and Skoog) MS media containing 6-benzyl amino purine (BAP) (2 mg/1), Naphthaleneacetic acid (NAA) (0/1 mg/l), selection of transformed explants was performed on MS medium supplemented with a BAP (2 mg/1), NAA (0/1 mg/l), cefotaxime (250 mg/1), and kanamycin sulfate (100 mg/1). The regenerated shoots were rooted on Indole*-3-*butyric acid (IBA) (1 mg/1), containing kanamycin sulfate (100 mg/1) and cefotaxime (250 mg/1). Afterward, seedlings were transferred into pots and grown in greenhouse conditions. Tobacco plants transformed with the pBI121 binary vector were used as a control in the experiments. Seeds of transgenic lines in T0 generation were selected in selection media containing 150 mg/l kanamycin sulfate. These plants were subsequently analyzed by PCR. The T1 PCR positive lines were selected and brought to T2 generation. The last step was repeated on T2 generation lines to gain homozygote seeds.

### PCR analysis

DNA was extracted using the CTAB 2% method from three generations of leaves of putative transgenic plants [41]. Primers were designed by Primer 3 online software [42], then synthesized by Gene script company (China). PCR was performed by 10X BIO FACT master mix (Korea), and the PCR products were loaded on agarose gel 1%. Then the gel image was captured by Gel document. PCR analysis was conducted in a reaction containing primers (S1 Table).

### Histochemical GUS assay of SBP-GUS

The youngest leaf (in T0, T1, and T2 generations), sepal, stamen, petal (T1 generations), and seeds (in T1, and T2 generations) were sampled at different stages of the vegetative and reproductive phases.

These tissues in different stages and generations of SBP-GUS, 35S-GUS, and non-transgenic plants, were prepared and assayed as described by Kosugi method. [43]. Tissues were photographed by microscopy.

### RNA isolation and real-time PCR

Seeds and leaves of one SBP-DGAT1 event and non-transgenic lines were prepared at the final phase of seed development. They were instantly placed in the liquid nitrogen and stored at—80°C. In the next step, the frozen tissue was ground under liquid nitrogen using a grinder (Geno Grinder, USA). Isolation of total RNA from seeds and leaves was carried out using Trizol reagent genomic. DNAs were removed via a DNase І treatment. The quality of isolated RNA was determined by electrophoresis on the agarose gel. The quantity of total RNA was finding out by the Nano Photometer apparatus (IMPLEN).

### cDNA preparation and qPCR reactions

Revert Aid First Strand cDNA Synthesis Kit (Fermentase) was utilized for synthesizing cDNA. Quantitative Real-Time PCR (qPCR) experiments were implemented and replicated three times using BIO FACT real-time master mix. Primers were designed by Primer 3 online software [42] and synthesized by Gene Script Company. Primer’s properties were summarized in the S2 Table. The primer specificity was confirmed using Primer blast in NCBI (https://www.ncbi.nlm.nih.gov). The qRT-PCR reaction was made with 5μl of diluted cDNA, 10 μl of 2X PCR Master Mix, and 1 μl of each primer (10 pmol). The final volume reached 20 μl with double distilled water. The PCR program consisted of several steps: 5 min at 94˚C, 40 cycles of 10 sec at 95 ˚C, 60 sec at 49 ˚C, 10 sec at 72 ˚C, and a final step of 30 sec at 72 ˚C. A melting curve analysis including 81 cycles at 55–95 ˚C with 0.5˚C increases in each cycle was performed to evaluate the primer specificity. The real-time PCR was done using the Roche Real-time system. Relative expression ratios were calculated through the comparative ΔΔC_T_ method by REST software for relative gene quantification, according to equation 1. A housekeeping gene (in this study, Actin) was applied as a reference gene with equal transcripts in all stages and tissues.


$$\mathrm{Ratio}=(E_{\mathrm{target}}{)}^{\mathrm{\Delta CP}\;\mathrm{target}\;(\mathrm{control}‐\mathrm{sample})}/(E_{\mathrm{ref}}{)}^{\mathrm{\Delta CP}\;\mathrm{ref}\;(\mathrm{control}‐\mathrm{sample})}$$

### Oil content analysis

Fifteen’s grams of seed and leaf from replications of three SBP-DGAT1 events, were ground, then moved into pre-weighed Whatman bags (Weight A) and sealed. These samples were oven-dried until reaching constant weight (Weight B). After that, the bagged sample was transferred into a Soxhlet tube and extracted with 120 ml petroleum ether for 24 h. The solvent was evaporated by heat, and after condensation was poured on the sample by the upper part of the device (glass refrigerant). When the Soxhlet tank was complete, the solvent and liquid extracted (oil) were returned to the balloon through a thin glass siphon. This cycle was repeated for 4 hours until almost all oil was transferred to the solvent. After the extraction, the bagged sample was dried to evaporate the remaining petroleum ether (Weight C). The oil content (%) was determined by (B—C)/(B—A)* 100% [44] formula.

### Fatty acid measurement

Gas chromatography (GC) was used to determine the Fatty acid composition from replicates of three SBP-DGAT1 events. For this purpose, TAG is converted to methyl ester based on the INSO 14880 standard method entitled “Oilseeds—Oil extraction and preparation of triglyceride fatty acid methyl esters for analysis by GC” (rapid method). Twenty ml of iso-octane was added to 0.1 gr of the sample. Then, 0.1 ml of 2 M potassium hydroxide methanol solution was added to the test. The lid was placed, and the sample was shaken vigorously for 1 minute. The solution was allowed to stand for 2 minutes. Subsequently, 2 ml of sodium chloride solution was added, and the mixture was shaken again. The iso-octane layer was dried over sodium hydrogen sulfate and injected into the GC (Agilent 7890A GC system. Carrier gas: Nitrogen. Inlet temperature: 220°C, Split ratio: 70:1. Detector (FID) temperature: 220°C. Injection volume: 0.4 μL. Colum oven: 170°C (5 min Hold) to 190°C Rate: 0.5°C/min. Column: DB WAX, 60 m, 0.25 mm (ID), film thickness: 0.25 μm) [45].

### Seed weight and size measurement

Mature seeds were harvested from non-transgenic (WT), and tobacco plants were grown under the same conditions. Three hundred seeds from non-transgenic and SBP-DGAT1.H1, F, and C2 tobacco plants in three replicates were oven-dried at 37°C for 24 h to ensure all the seed moisture content of samples was equal. The seeds were weighed by analytical balance. Mature seeds were photographed with a stereoscopic microscope (OPTIKA, ITALY), and the seed size was analyzed by Image J 1. X software.

### Statistical analysis

One-way ANOVA was used to compare six transgenic and non-transgenic plants for measurements of seed and leaves percentage oil content, FA composition, seed size, seed weight, and fold-change in gene expression. The values represent at least three biological replicates. A p-value < 0.01 was considered as significant. The individual lines were compared again by non-transgenic by LSD test as indicated in the fig legends using SAS (version 6.4) for the data with significant differences.

## Results and discussion

### In silico analysis of SBP promoter

According to SBP sequence findings, we didn’t find any homology in PlantPromDB (35), which indicates no data about SBP has been reported yet. However, database searching at PLACE revealed that SBP contains 37 diverse *cis-*acting elements, including: i) general transcription factors; ii) cis-acting in the regulatory element expressed in the seeds; iii) *cis*-acting in regulatory element related to abscisic acid (ABA); iv) *cis*-acting in the regulatory element in the TAG pathway indirectly, and v) other *cis*-acting regulatory elements. The *cis*-acting regulatory elements related to seed expression and tissue-specific expression are categorized in (Table 1).

**Table 1:** Identified *cis*-acting regulatory elements in SBP using PLANTCARE and PLACE databases.

Category	Factor or Site Name	Signal seq	Function	Position
***cis* -actin regulatory elements related to seed expression**				
**1- *cis* -acting in a regulatory element which expressed in the seed**	CEREGLUBOX2PSLEGA	TGAAAACT	Cereal glutenin box, homologous to the cereal Glutenin gene control element	208 (+)
	-300ELEMENT	TGHAAARK	Endosperm speci*fi*c	592 (+)
	GADOWNAT	ACGTGTC	GA-down regulated d1 cluster	285 (+)
	SEF4MOTIFGM7S	RTTTTTR	SEF4 binding site	321 (+), 1108 (+)
	CAREOSREP1	CAACTC	Gibberellin regulated proteinase expression	372 (+)
	NAPINMOTIFBN	TACACAT	Seed storage protein	1191 (-)
	AACACOREOSGLUB1	AACAAAC	Endosperm speci*fi*c	533 (+)
	RYREPEATBNNAPA	TTTTTTCC	RY repeat of ABA inducible RY/G box required for Seed speci*fic* expression	1644 (+), 1773 (-)
	GTGANTG10	GTGA	GTGA motif in late pollen gene g10	5 (+), 1025 (+), 1349 (+), 1356 (+)
	POLLEN1LELAT52	AGAAA	Pollen specific activation	141(+), 464 (+), 708 (+), 1010 (+), 1083 (+), 1209(+), 1246 (+), 171 (+)
**2- *cis* -acting in regulatory element related to ABA**	ABRERATCAL	MACGYGB	ABRE-related sequence	18 (+)
	ABRELATERD1	ACGTG	ABRE-like sequence; ABA and dark induced Senescence	7 (+), 205 (+), 285 (+)
	ACGTABREMOTIFA2OSEM	ACGTGKC	ACGT-core of motif A in ABRE. ABA- responsive expression	285 (+)
	MYBATRD22	CTAACCA	Binding site for MYB, ABA; MYC	228 (+)
**3- *cis* -acting in a regulatory element which is seed-specific and related to ABA.**	ABREMOTIFAOSOSEM	TACGTGTC	ABRE-like sequence	1147 (-)
	ACGTABREMOTIFAOSOSEM	TACGTGTC	ABRE motif A	1747 (-)
	EBOXBNNAPA	CANNTG	E-Box drive light-responsive expression, storage Protein	6 (+), 623 (+), 1350 (+), 1708 (+)
	PYRIMIDINEBOXHVEPB1	TTTTTTCC	GA induction GA; ABA; GARE; pyrimidine box; seed; aleurone	1183 (-)
	DPBFCOREDCDC3	ACACNNG	ABA inducible bZIP transcription factor DPBF-1 & 2 binding sites	1760 (+)
	MYCCONSENSUSAT	CANNTG	MYC recognition site found in the promoter of Dehydration responsive genes	6 (+), 623 (+), 1350 (+), 1708 (+)
	MYB2CONSENSUSAT	YAACKG	MYB recognition site	459 (+), 838 (-)
	MYB1AT	WAACCA	MYB recognition site found in the promoter of Dehydration responsive gene rd22	229 (+), 980 (+), 1086 (+)
**4- *cis* -acting in a regulatory element in TAG pathway indirectly**	GATABOX	GATA	GATA box light-regulated, and tissue-specific Expression	11 (+), 48 (+), 1059(+), 1392 (+),1434 (+)
	DOFCOREZM	AAAG	Core sequence of DOF transcription factor binding site, and endosperm specific	25 (+), 295 (+), 501 (+), 596 (+), 602 (+), 753 (+), 896 (+), 937(+), 1062 (+), 1226 (+), 1577 (+), 1638 (+)
	CACGTGMOTIF	CACGTG	G-box, essential for expression of beta- phaseolin gene during embryogenesis	6 (+)
**Other important *cis*-acting regulatory elements**				
**Tissue/ organelle specific expression**	CACTFTPPCA1	YACT	Mesophyll speci*fi*c expression in C4 plants	13 (+), 179 (+), 269 (+), 802 (+), 876 (+), 882 (+), 890 (+), 987 (+), 113 (+),1386 (+),1618 (+), 1725 (+)
	BOXIINTPATPB	ATAGAA	Box II motifs on some non-consensus type plastid Promoters	1207 (+), 1244 (+)
	NODCON2GM	CTCTT	Nodule speci*fi*c expression	295 (+)
	OSE2ROOTNODULE	CTCTT	Nodule and organ speci*fi*c expression after infection	181 (+), 375 (+), 638 (+), 1280 (+), 1316 (+), 179 (+)
	RHERPATEXPA7	KCACGW	Root hair speci*fi*c expression	5 (+)
	S1FBOXSORPS1L21	ATGGTA	S1F box; repressor of plastid ribosomal protein S1 and L21	1660 (+), 1711 (+), 1216 (+)
	TAAAGSTKST1	TAAAG	Guard cell speci*fi*c expression mediated by Dof1 Protein	24 (+), 936 (+), 1061 (+), 1576 (+)

We have found 22 sequences that are expressed in the seeds directly. However, some *cis*-elements contribute to seed-specific expression or the TAG pathway, indirectly. GATABOX interacts with HvMYBS3 in yeast three-hybrid assays, and HvMYBS3 enhances the expression of the developing endosperm-specific gene in barley seeds [46]. According to sequence searching, the GATA motif was repeated three times in the top strand of SBP between 1000 and 1378 bp.

DOFCOREZM, as a member of DOF proteins by AAAG sequence, regulates the expression of genes that correlate with carbon metabolism in maize. We found this motif in SBP with 12 repeats in the + strand and 19 repeats in the–strand, which indicates the importance of this motif in carbon metabolisms [47].

CACGTG MOTIF, or G-box was found in the SBP sequence of six bp position in both strands of our studies which was reported seed-specific expression in *Catharathus*. *roseus* strictosidine synthase gene promoter controls CACGTGMOTIF [48]. Moreover, such motif with a consensus sequence of CACGTG, was found in various plants in *RbcS* promoters [49].

Besides, by *cis*-element searching on Napin sequence as a strong seed-specific promoter, we observed eight seed-specific *cis*-elements identical to those in SBP include: MYCCONSENSUSAT, MYB1AT, SEF4MOTIFGM7S, DPBFCOREDCDC3, MYB2CONSENSUSAT, NAPINMOTIFBN, RYREPEATBNNAPA, and EBOXBNNAPA, which have been explained previously.

Furthermore, the AACA motif [50], GCN4 motif [51], Skn-1-like motif, RY repeat, and G-box [52] are critical seed-specific elements. Yuan et al. analyzed 108 Seed-Specific Candidate Genes (SSCGs) in peanut, 94 of which was expressed only in the seed. In addition, the expression of 14 of these SSCGs preferentially occurred in the seed [8]. It is accepted that RY REPEAT and GCN4 are conserved among many SSPs. This motif is conserved in seed promoters of legumin, essential for tissue-specific expression [53]. Moreover, we found the AACA motif in 474 bp in + strand. There are RY repeat and G-box in SBP, too. As discussed, many of the *cis*-elements in SBP were reported in other plants as seed-specific *cis*-regulatory elements.

Moreover, we demonstrated other *cis*-elements in response to environmental and hormone signals like ethylene-responsive element, light-responsive *cis*-acting element, anaerobic induction elements, defense, stress responsiveness elements, and salicylic acid responsiveness element.

### Functional analysis of SBP promoter

#### Generation of transgenic plants

**Fig 2:**
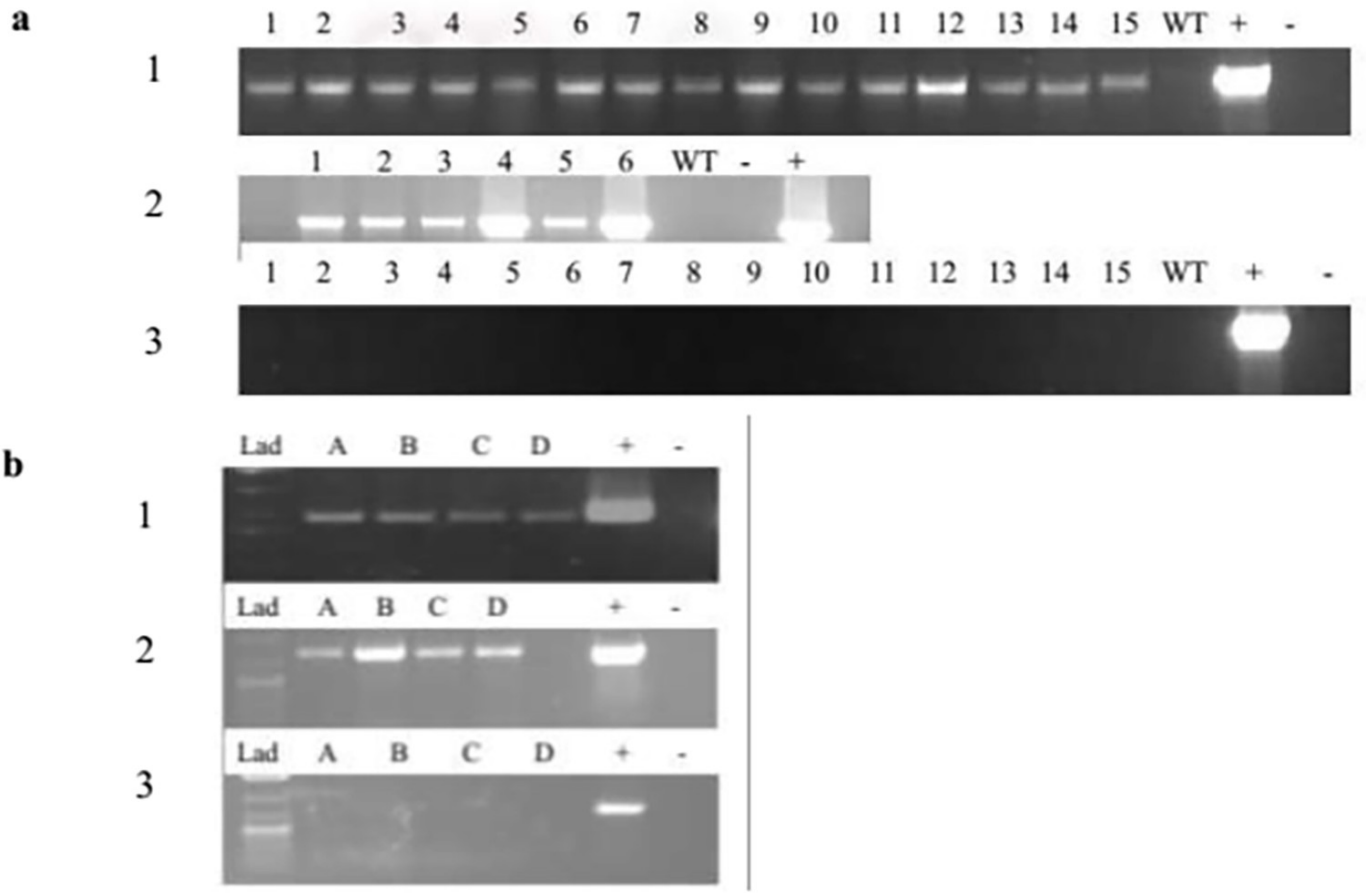
Detection of the transgenic tobacco by PCR analysis: **a.** SBP-DGAT1 transgenic plants; 1: DGAT1 (1135 bp), 2: nptII (1400 bp), and 3: vir (900 bp) are the PCR products that were synthesized by DGAT1, nptII, and vir forward and reverses primers. 1–15: putative SBP-DGAT1 transgenic plants, WT: non-transgenic plant, (+): positive control, (-): negative control. **b.** SBP-GUS transgenic plants; 1: SBP (574 bp), 2: nptII (700 bp), and 3: vir (700 bp) are the PCR products that were synthesized by SBP, nptII, and vir forward and reverses primers. A- D putative SBP-GUS transgenic plants, (+): positive control. (-): negative control.

PCR analysis confirmed the presence of *sbp* and *nptII* genes in the SBP-GUS putative transgenic plants and *nptII* and *DGAT1* genes in SBP-DGAT1 putative transgenic plants. Moreover, the absence of remaining bacteria was confirmed when amplification with *vir* specific primers was not detected in the DNA of transgenic plants (Fig 2) (S1 Raw images). Putative transgenic tobacco plants were selected on selection media containing kanamycin, and analysis of PCR amplification products revealed in all cases the presence of DNA encoding kanamycin resistance genes in all transgenic lines and its absence of untransformed negative control, indicating the selection procedure with kanamycin was optimal. Moreover, transgenic plants were transferred into the soil, and grown under greenhouse conditions to generate seeds.

#### Analysis of GUS gene expression in SBP transgenic tobacco plants

**Fig 3:**
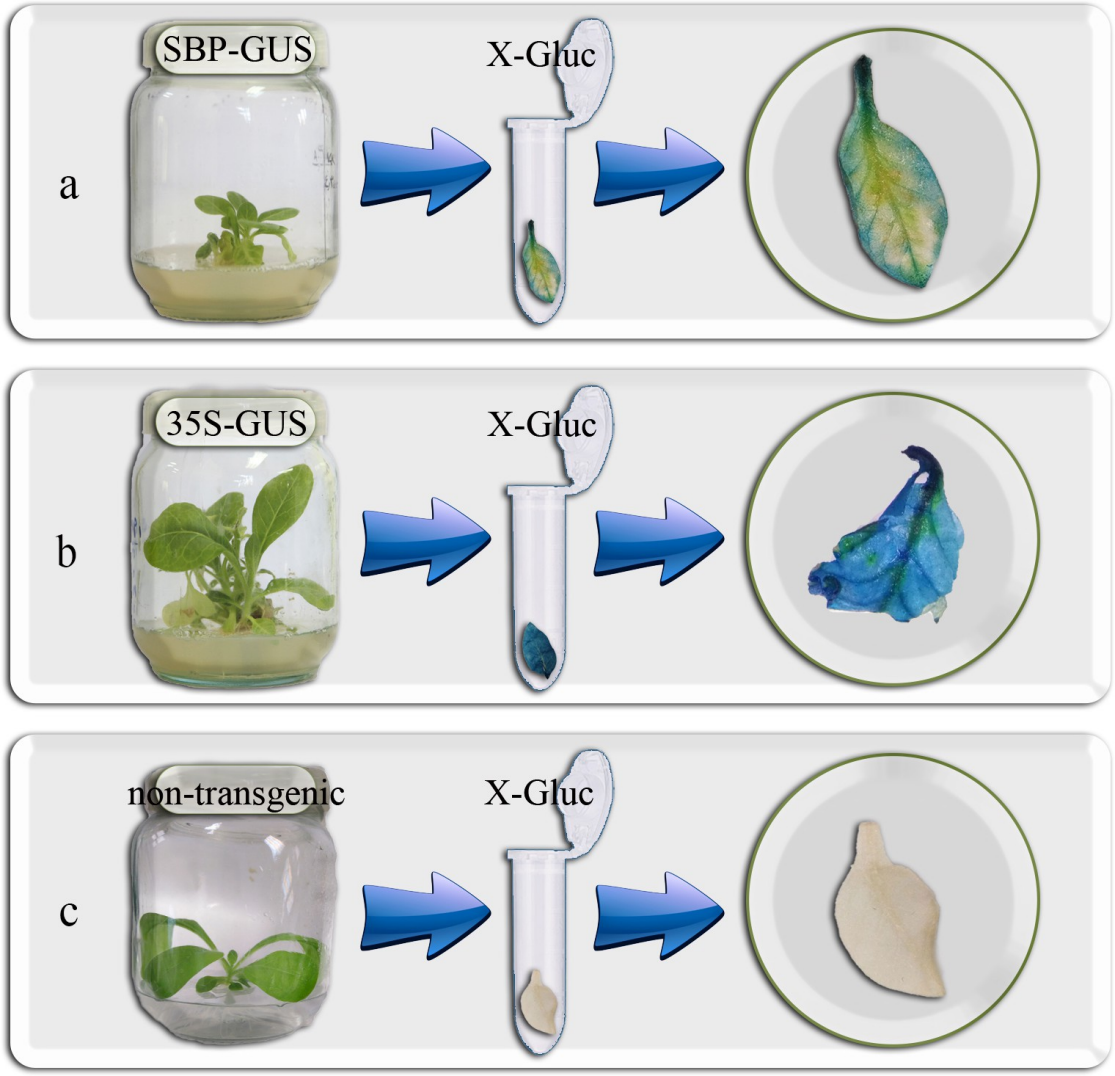
Histochemical GUS analysis of transgenic tobacco plants at T0 generation: **a**. transgenic plant **b**. 35S-GUS **c.** non-transgenic control.

Histochemical GUS assay was conducted on the leaves of T0 transgenic tobacco for SBP-GUS, 35S-GUS, and non-transgenic plants (Fig 3). As shown in Fig 3, the leaves were stained partially in T0 transgenic SBP-GUS plants, which indicates successful pSBP-GUS transfer and expression (Fig 3A). However, 35S-GUS, which has a constitutive promoter (CaMV 35S), was stained completely (Fig 3B), and there was no GUS activity in the non-transgenic plants (Fig 3C).

**Fig 4:**
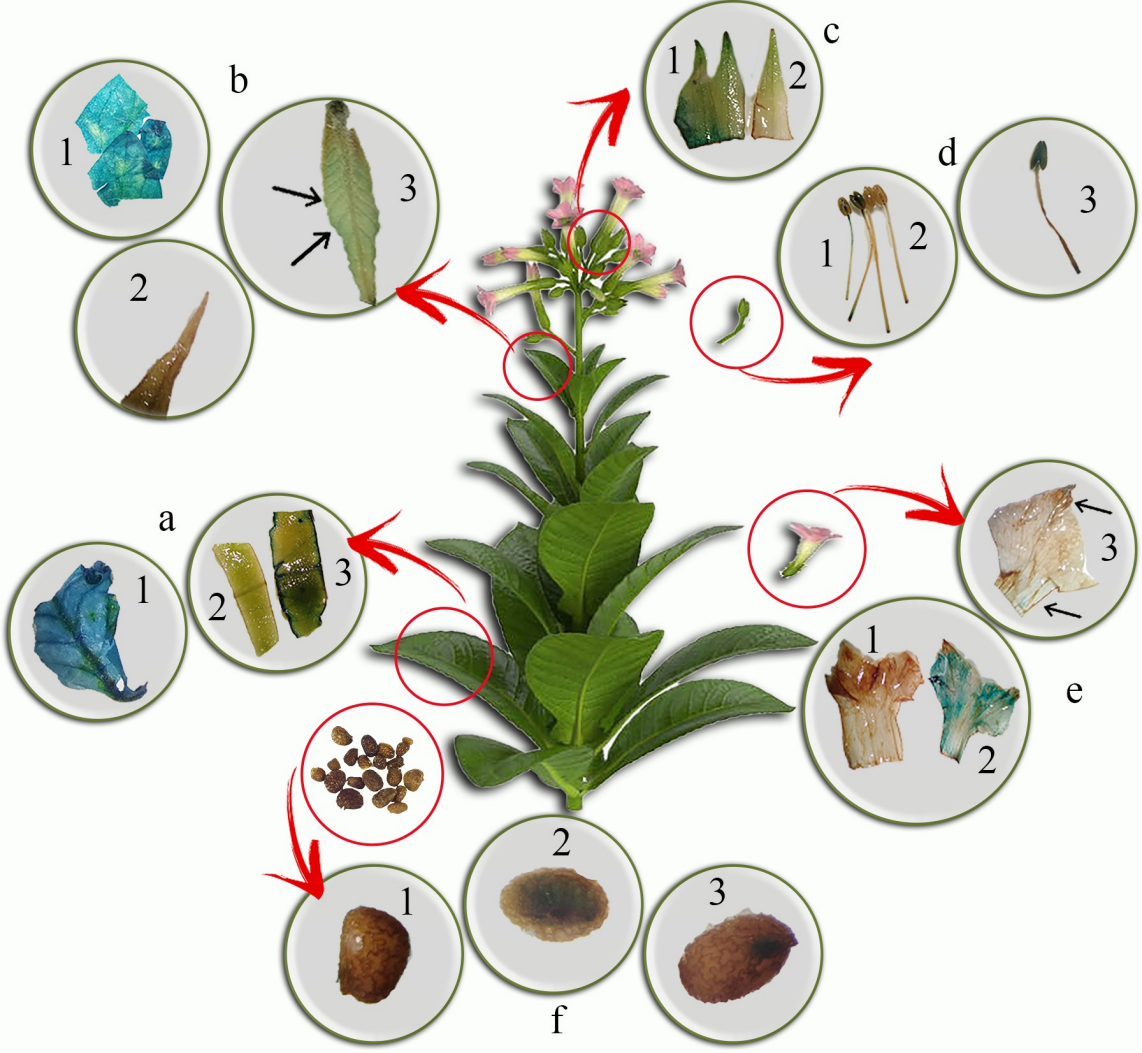
Histochemical GUS analysis of T1 generation of SBP-GUS transgenic tobacco plants. **a.** vegetative leaf **b.** reproductive leaf **c.** sepal **d**. stamen **e.** petal **f.** seeds: a.1, b.1, d.1, e.2, and f.2 represented GUS analysis in 35S-GUS samples. a.2, b.2, c.2, d.2, e.1, f.1 represented GUS analysis in non-transgenic plants. a.3, b.3, c.1, d.3, e.3, and f.3 represented GUS analysis in SBP-GUS samples.

T1 generation of putative SBP-GUS transgenic lines was stained by X-Gluc in different developmental stages and organs. The results indicated GUS activities are differentially in the vegetative and reproductive stages of transgenic (Fig 4). GUS assay was used for staining the transgenic SBP-GUS leaves in the vegetative state (90 old days), and little GUS expression was observed in 90 old days leaves (Fig 4A.3). However, there was no GUS activity in non-transgenic leaves (Fig 4A.2), and leaves of 35S-GUS were stained completely (Fig 4A.1).

However, the GUS activity in SBP-GUS decreased from the reproductive to the flowering stage. In the flowering stage, leaves that previously showed GUS activity (Fig 4A.3), almost underwent ceased GUS activity in transgenic SBP-GUS (Fig 4B.3). These decreased changes in gene expression in the same tissue were reported in *OsRGLP2* expression in tobacco [54]. However, GUS activity was identified in reproductive organs, including sepal (Fig 4C.1), stamen (Fig 4D.3), petal (Fig 4E.3), and immature seeds in SBP-GUS lines (Fig 4F.3). The GUS activity in 35S-GUS transgenic plants as positive controls was stable in all stages and tissues (Fig 4A, 4B, 4D.1 and 4E, 4F.2).

**Fig 5:**
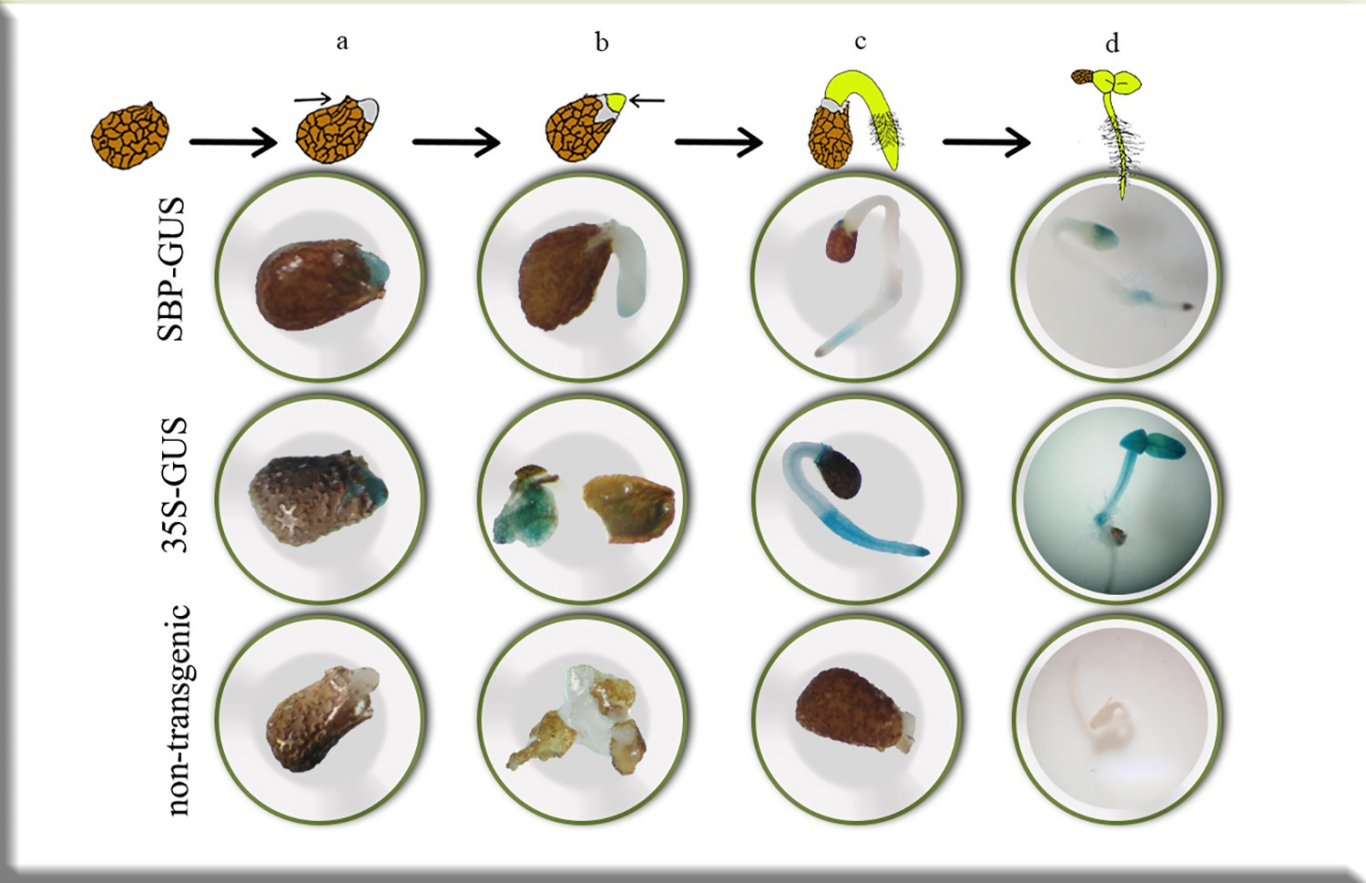
Histochemical GUS analysis in four stages of seed germination: **a**. endosperm appearance **b**. radical emerges **c**. radical growth and **d**. cotyledon appearance in SBP-GUS, 35S- GUS, and non-transgenic control plants.

To ensure the activity of the SBP promoter in the seed, a histochemical test was performed at various stages of germination in seeds. The present results demonstrated the SBP promoter activity at different stages of seed germination (endosperm appearance, radical emergence, radical growth, and, cotyledon appearance) in SBP-GUS transgenic plants (Fig 5). Moreover, GUS assay analysis indicated SBP promoter activity in the seed’s endosperm as the oil source of transgenic tobacco. However, SBP activity showed a decreasing pattern during the leaf development stages of SBP-GUS transgenic (Fig 4A.3 and 4B.3). The GUS activity was observed in the all-developmental stages of 35S-GUS plants (Fig 4A.1, 4B.1, 4D.1, 4E.2 and 4F.2).

**Fig 6:**
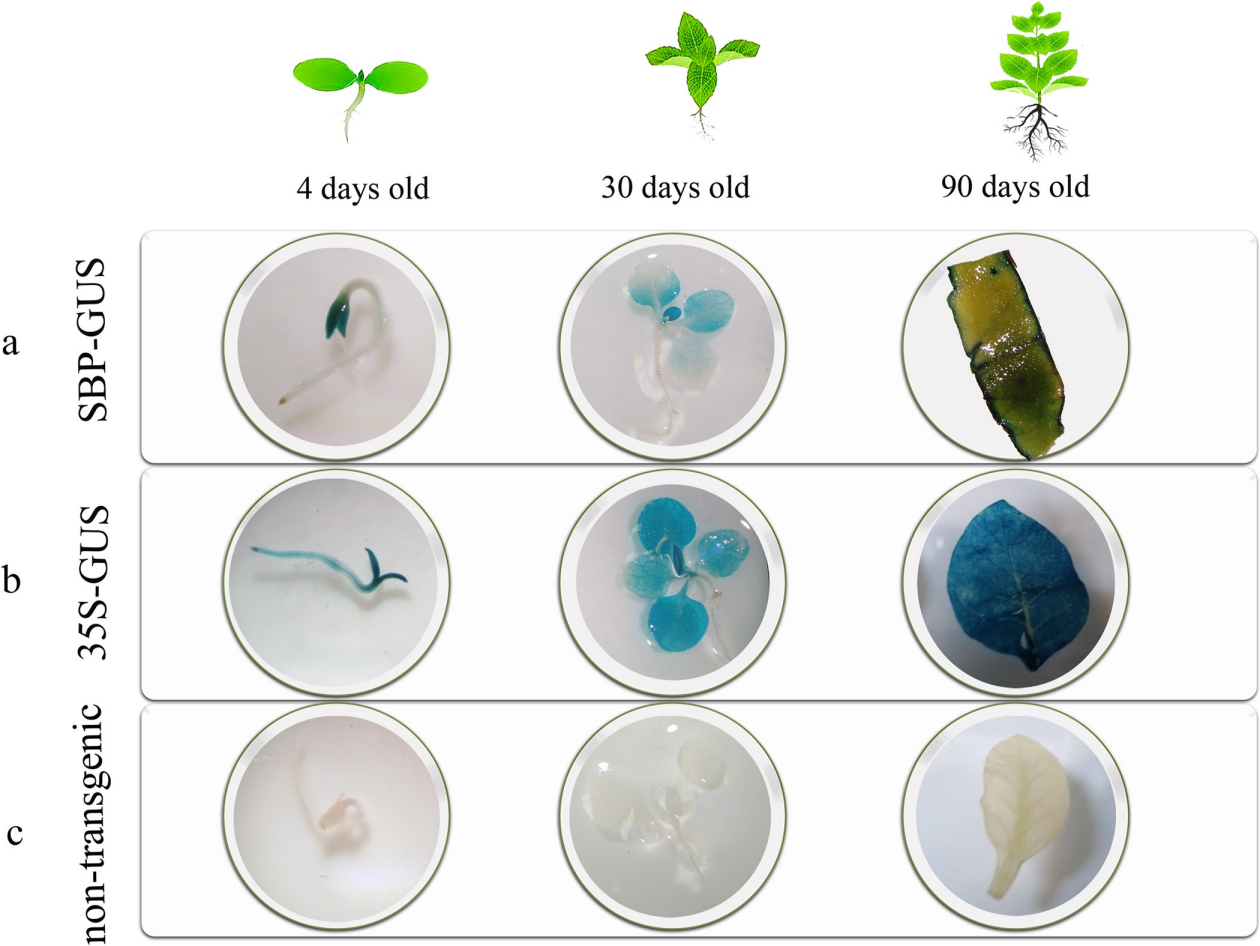
Histochemical GUS analysis in different stages of plant growth (4, 30, and 90 old days) in: **a**: SBP-GUS, b. 35s-GUS, and c. non-transgenic control plants.

Transgenic T1 generation seeds (SBP-GUS and 35S-GUS) were selected on media containing kanamycin for achieving the T2 generation lines. T2 generation of transgenic SBP-GUS, 35S-GUS, and wild-type plants, were investigated in three developmental stages (4-, 30-, and 90-days old seedlings after germination) using GUS-assay analysis (Fig 6). As expected, GUS activity in 90-day old transgenic SBP-GUS seedlings was less than that in four-day-old seedlings. However, GUS activity showed a steady-state in transgenic 35S-GUS in the three stages (Fig 6).

Various reports have been presented on the different expressions of the *SBP* gene in other plant tissues. Bahry and Zimmer (2016) reported a higher level of SBP expression in the initiation stage of seed coat development of soybean [55]. Moreover, in tobacco plants, SBP activity is indicated in the microsomal fraction of young leaves [56]. The expression of β-glucoronidase (GUS) and green fluorescence protein (GFP) genes under the control of soybean GmSBP2/S64 were explicitly reported in the phloem of leaves, stems, and roots of transgenic tobacco plants [57]. Despite similar structures of SBP genes, their function has been reported differentially in various plants.

While soybean SBP accelerates sucrose uptake, *VfSBPL* expression in yeast and transgenic potato plants did not affect in sucrose transport and carbohydrate status, respectively [58]. Interestingly, *VfSBPL* was expressed in seeds while *GmSBP1* transcript levels were confirmed in young sink leaves [18].

The present functional analysis of the SBP promoter confirmed the preliminary results of the sequence analysis on the specific activity of the SBP promoter in the plant seeds.

#### DGAT1 gene expression analysis

**Fig 7:**
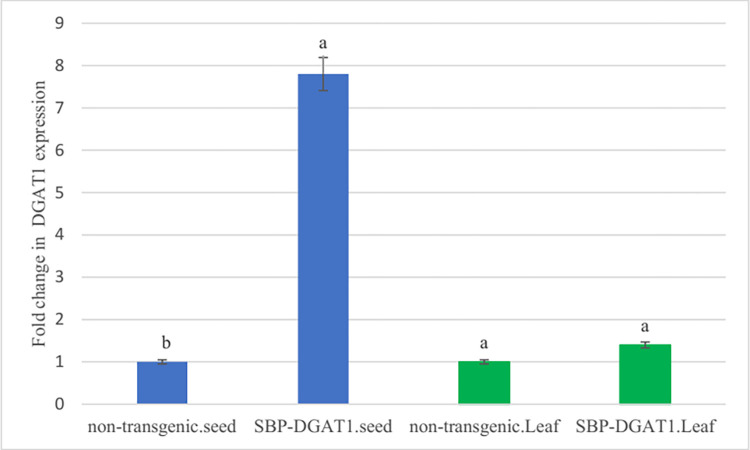
Functional impact of SBP promoter on mRNA levels of *DGAT1* in seed and leaf of the transgenic plant (SBP-DGAT1). Relative expression levels are expressed as fold changes in blue bars: seeds and in green bars: leaves. Mean values with different letters are significantly different by one-way ANOVA (P < 0.01), n = 6.

DGAT1 expression was measured by real-time PCR. The results showed a higher gene expression level in seeds of transgenic plants in T3 generation by 7.8-folds more than in control plants (Fig 7). These results are consistent with other studies that have increased *DGAT* gene expression under the control of seed-specific promoters [59, 60]. Also, there is no difference in the *DGAT* gene expression of leaves.

#### Oil content analysis

**Fig 8:**
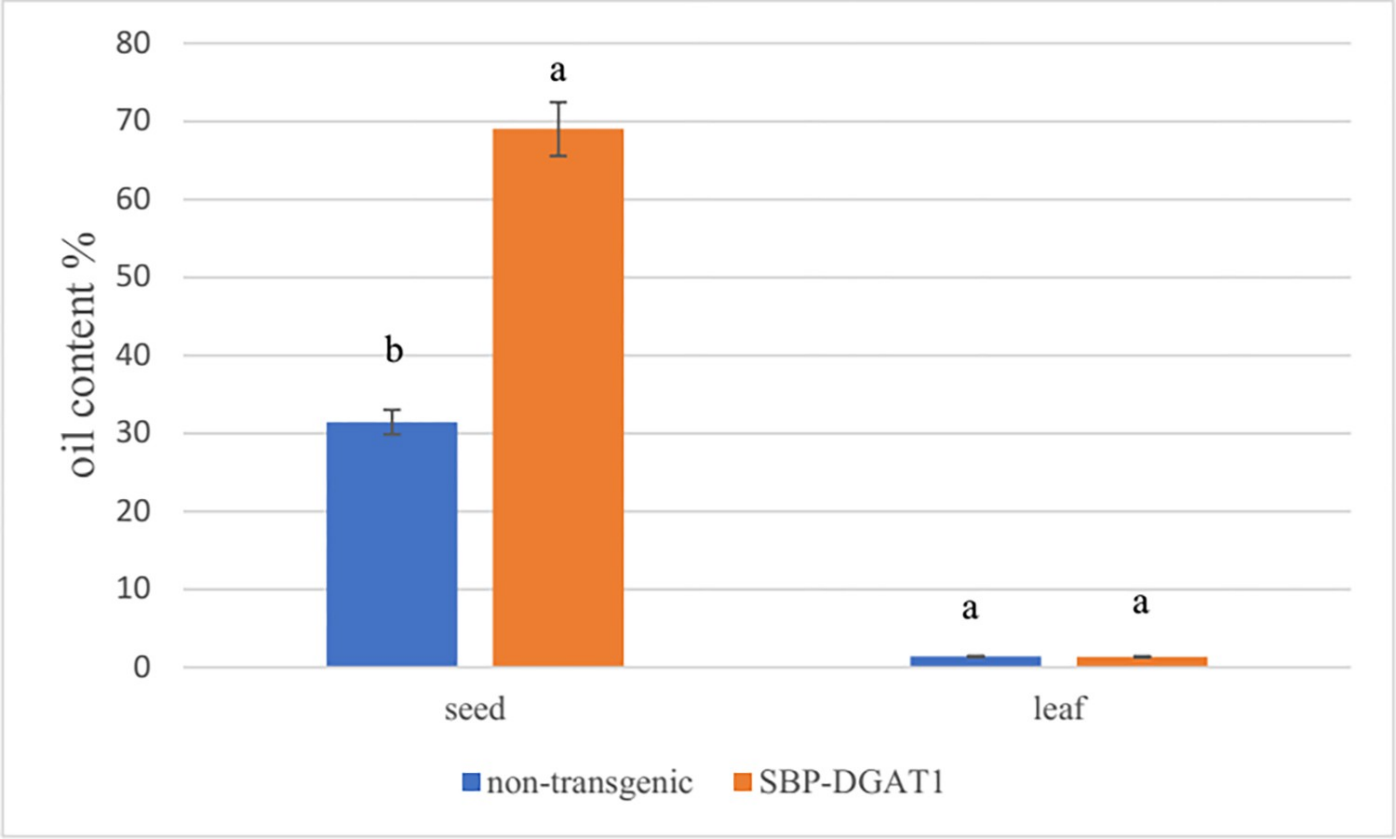
Functional impact of SBP promoter on the seed and leaf oil content (%) of transgenic (SBP-DGAT1) and non-transgenic tobacco lines. Mean values with different letters are significantly different by one-way ANOVA (P < 0.01), n = 6.

The seed and leaf oil content (based on dry weight) of independent transgenic lines (T3 generation) and SBP-DGAT1 transgenic lines were analyzed as described in the material and method section. The average oil content in non-transgenic tobacco plants was 31.43%. However, it was 68.99% for SBP-DGAT1 transgenic lines, which showed 2.19 times more than non-transgenic lines (Fig 8). As shown in Fig 8, the oil content in the leaves of the transgenic plant and the control plant is no different.

Transferring the *DGAT1* gene under the control of the Oleosin promoter in *Arabidopsis* led to an increase in oil content up to 8.3% compared to the wild-type plants [32]. The simultaneous down regulation of the *DGAT1* gene under the GmUbi3 promoter led to a reduction in oil content in soybean to 8.3% [61]. Moreover, *AtDGAT1* expression under the control of Napin, as a seed-specific promotor, showed an 11–29% increase in oil content in transgenic *Arabidopsis* plants [31]. The expression of *DGAT1* allele, PH09B, in the maize by controlling of an oleosin promoter resulted in an increase in kernel oil of about 1% in transgenic maize plants [62]. Moreover, overexpression of *AtDGAT1* in *B*. *napus*, under the control of the Napin promoter, increased the seed oil content by 2.5% to 7% [26].

#### Fatty acid analysis

**Fig 9:**
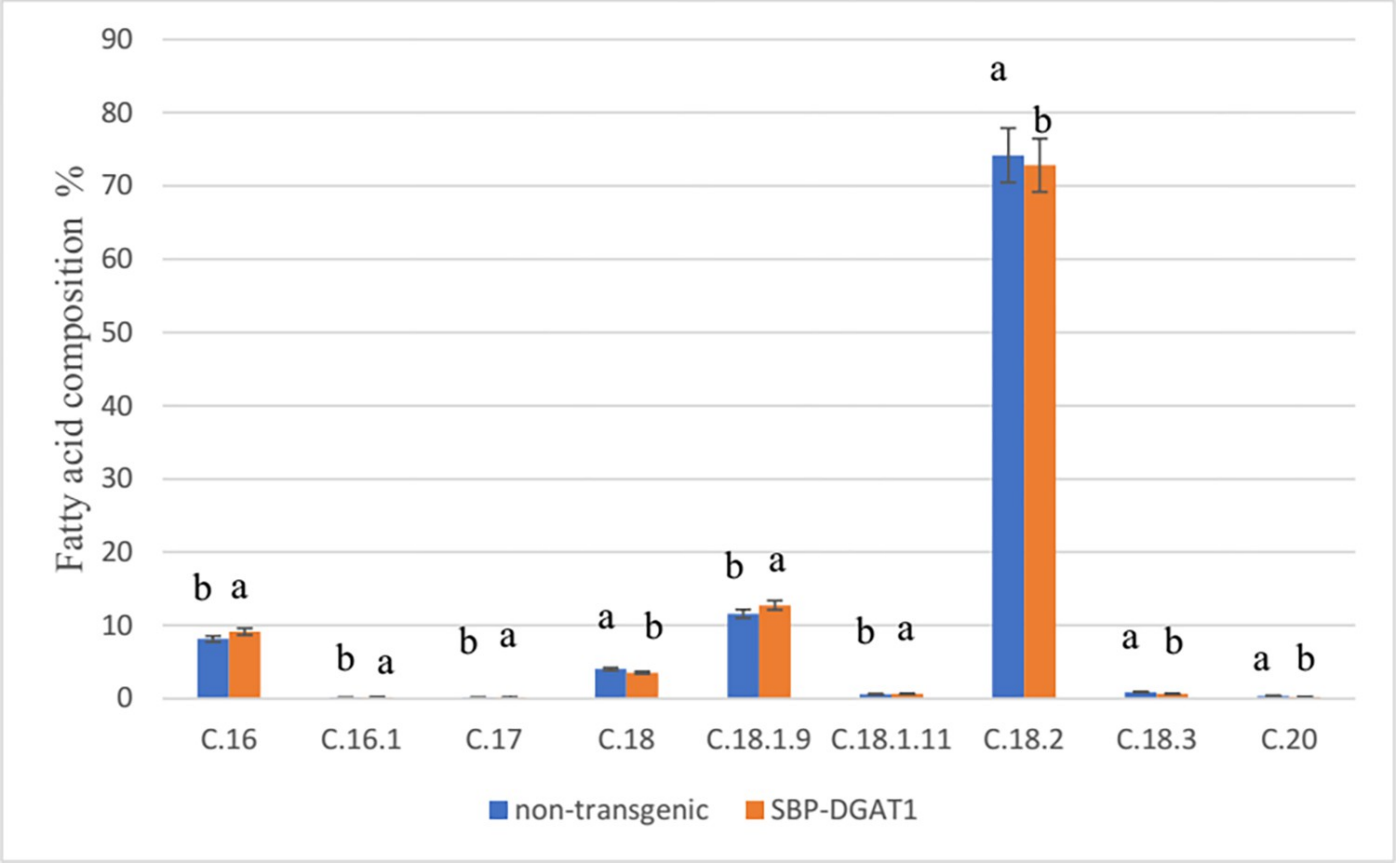
Functional impact of SBP promoter on fatty acid composition in SBP-DGAT1 transgenic plant seeds. Mean values with different letters are significantly different by one-way ANOVA (P < 0.01), n = 6.

The fatty acid profiles of the seed oil in transgenic SBP-DGAT1 plants were determined by GC analysis. We have found nine fatty acids (FAs). Nine principal fatty acids were Palmitic acid (16:0), Palmitoleic acid (16:1), Margaric acid (17), Stearic acid (18:0), Oleic acid (18:1.9), Vaccenic acid (18:1.11), Linoleic acid (18:2), Linolenic acid (18:3), and Arachidic acid (20:0). According to GC results, the expression of the *DGAT1* gene, driven by the SBP promoter, cause a significant increase in the content of Palmitic acid, Palmitoleic acid, Margaric acid, Oleic acid, Vaccenic acid by 12.1%, 33.9%, 10.79%, 10.16%, and 5.7% respectively. However, Stearic acid, Linoleic acid, Linolenic acid, and Arachidic acid contents decreased to 12.39%, 1.83%, 28.6% and, 38.1%, respectively (Fig 9).

The fatty acid profiles of seed oils in oilseed crops are characterized by three fatty acids: Palmitic acid, stearic acid, Oleic acid, and Linoleic acid [63]. Oleic acid is a monounsaturated fatty acid vital for fighting pathogens, transporting minerals, and responding to hormones. Oleic acid also serves as the most important energy source for our cells, and it’s used for the production and biosynthesis of many crucial metabolites [64]. It was shown that Oleic acid is beneficial both in the immunomodulation, treatment, and inhibition of different types of disorders such as cardiovascular or autoimmune diseases, metabolic disturbances, skin injury, and cancer [65–67]. Moreover, promoting Oleic acid content and reducing Linolenic acid content led to decreasing undesirable trans-fats in oils [61, 68]. According to our results, Oleic acid content increased by 10.16%, and Linolenic acid content decreased by 28.6% in SBP-DGAT1 transgenic plants (Fig 9).

#### Seed size and weight

**Fig 10:**
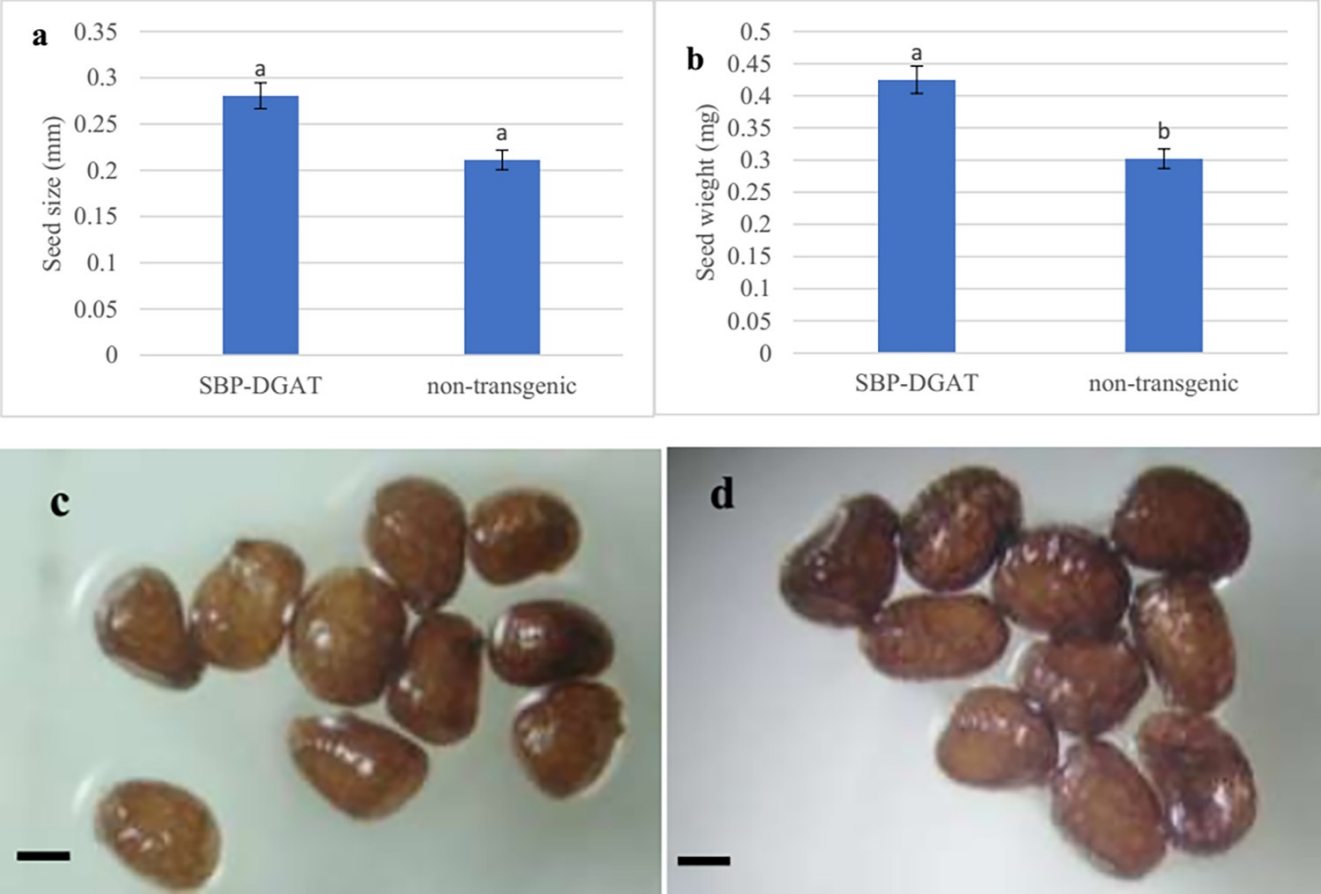
Functional impact of SBP promoter in size and weight of transgenic seeds: **a.** seed size (mm) and **b.** seed weight (mg). Seed’s size in **c.** non-transgenic and **d.** SBP-DGAT1 plants. Bars = 1mm. Mean values with different letters are significantly different by one-way ANOVA (P < 0.01), n = 6.

Transgenic seeds showed no significant changes in size compared to non-transgenic seeds (Fig 10A, 10C and 10D). Our finding is according to the previous reports [69] and [32]. Nevertheless, despite non-significant changes in the size of transgenic seeds of plants, the weight showed a significant increase than the non-transgenic control plants. (Fig 10B). These results agree with the results of two other studies in the *DGAT1* gene [61, 70]. A survey of the *Arabidopsis* transgenic plants by *DGAT1* showed a 3.5%–10% increase in seed weight and total seed yield in transgenic lines [70]. Moreover, Torabi et al. reported that *DGAT1* soybean transgenic lines showed a significant increase in seed weight than the control plants [61].

## Conclusion

In the current study, we proposed SBP as a seed-specific promoter to prompt the oil content in oilseed crops. The SBP promoter was examined in the presence of seed-specific motifs and *cis-*elements. SBP-GUS construct was transferred to the tobacco plant as a model plant. The activity of the SBP promoter in different stages, from germination to the seedling stage was investigated. After introducing *DGAT*, which is regulated by SBP promoter, the oil content and composition of FA were evaluated.

As a result, the maximum activity of the SBP promoter could probably be observed in the seed stage of oil plants, which is desirable for seed-specific expression of oil metabolism-dependent genes. The expression of the *DGAT* gene in the seeds of transgenic plants was 7.8-fold higher than of control plants. On the other hand, the expression of this gene in the leaves of transgenic plants did not show any change compared to control plants. These results also indicate more effect of the promoter on the seed than other tissues. Based on our results, SBP as a seed-specific promoter can be exploited as a novel promoter for over-expression of essential genes in oilseed crops in favor to increase oil and oleic acid content, as essential seed traits for getting better the quality of oil which have various usage in food, medicinal and industrial.

In conclusion, we revealed that the effect of SBP on *DGAT1* expression and accumulation of seed oil content is significant. Also, the oil content in the leaves did not show any significant changes. In addition, we have found a noticeable increase in Oleic acid and decreasing in Linolenic acid contents in the transgenic lines. Additional studies can help to understand the function of this promoter better. And transferring the genes related to TAG pathway under the control of SBP promoter and analysis of changes in the oil content in various tissues in tobacco and oily plants are suggested to understand the function of this promoter. Reporting any firm conclusions requires other well-designed research and experiments. This was an attempt to open the way for future investigations.

## Supporting information

S1 Table Oligonucleotide sequences used for genomic DNA PCR. (DOCX)

S2 Table Oligonucleotide sequences used in quantitative real-time PCR. (DOCX)

S1 Raw images Detection of the transgenic tobacco by PCR analysis. (PDF)
