# Supplementary material for: Characterization of sucrose binding protein as a seed-specific promoter in transgenic tobacco Nicotiana tabacum L
Source: PLoS One. 2022 Jun 3;17(6):e0268036. doi: 10.1371/journal.pone.0268036 (PMC9165846; doi:10.1371/journal.pone.0268036)
Supplement: S1 Table — (DOCX) [file pone.0268036.s001.docx]

**S1 Table**

Oligonucleotide sequences used for genomic DNA PCR

| Forward primer | Reverce primer | Primer name |
| --- | --- | --- |
| ACCAACTGTCTAACAAC | TATGCCTGAATCTACTG | DGAT1 |
| CTCACCTTGCTCCTGCCGAGA | CGCCTTGAGCCTGGCGAACAG | nptII-215 |
| AAACTCTGATTGAACCTAC | GGATTCTTTGTGTTGTAC | SBP |
| ATGATTGTACATCCTTCACG | TGCTGTTTTTATCAGTTGAG | virG |
