# Supplementary material for: Characterization of sucrose binding protein as a seed-specific promoter in transgenic tobacco Nicotiana tabacum L
Source: PLoS One. 2022 Jun 3;17(6):e0268036. doi: 10.1371/journal.pone.0268036 (PMC9165846; doi:10.1371/journal.pone.0268036)
Supplement: S2 Table — (DOCX) [file pone.0268036.s002.docx]

**S2 Table**

Oligonucleotide sequences used in quantitative real-time PCR

| Gene name | Primer name | Tm | GC% | Sequence | Product length (bp) |
| --- | --- | --- | --- | --- | --- |
| *DGAT1* | CaD. For | 45.1 | 35.3 | 5-' TGAATCTACTGGAACTA -3' | 168 |
|  | CaD. Rev | 47.0 | 33.3 | 5-' GAATCTCTAACTCTAACA3 -3' |  |
| *Actin* | Actin. For | 59.73 | 52.38 | 5-' CATTGTGCTCAGTGGTGGTTC -3' | 200 |
|  | Actin. Rev | 60.00 | 52.38 | 5-' TCTGCTGGAAGGTGCTAAGTG-3' |  |
