# Supplementary figures and images for: Characterization of sucrose binding protein as a seed-specific promoter in transgenic tobacco Nicotiana tabacum L
Source: PLoS One. 2022 Jun 3;17(6):e0268036. doi: 10.1371/journal.pone.0268036 (PMC9165846; doi:10.1371/journal.pone.0268036)

a. 1

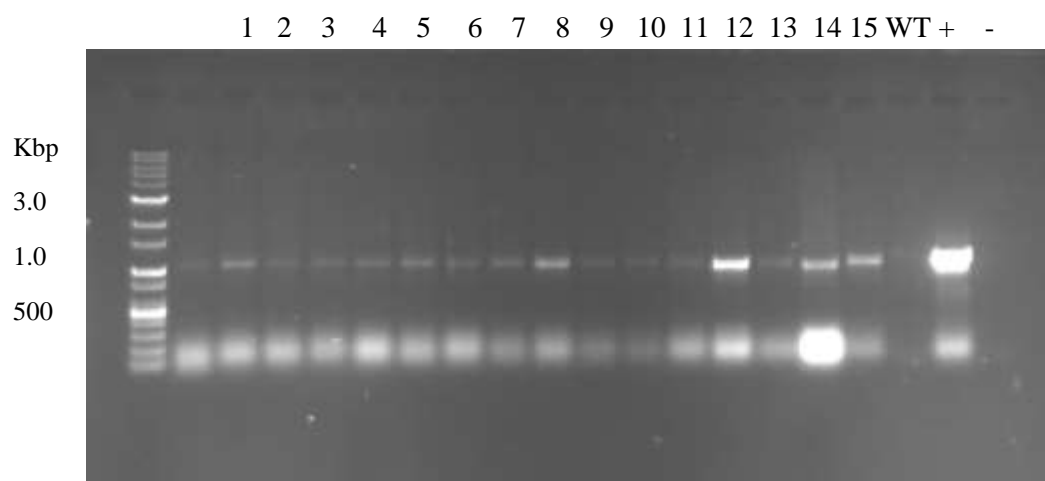

a. 2

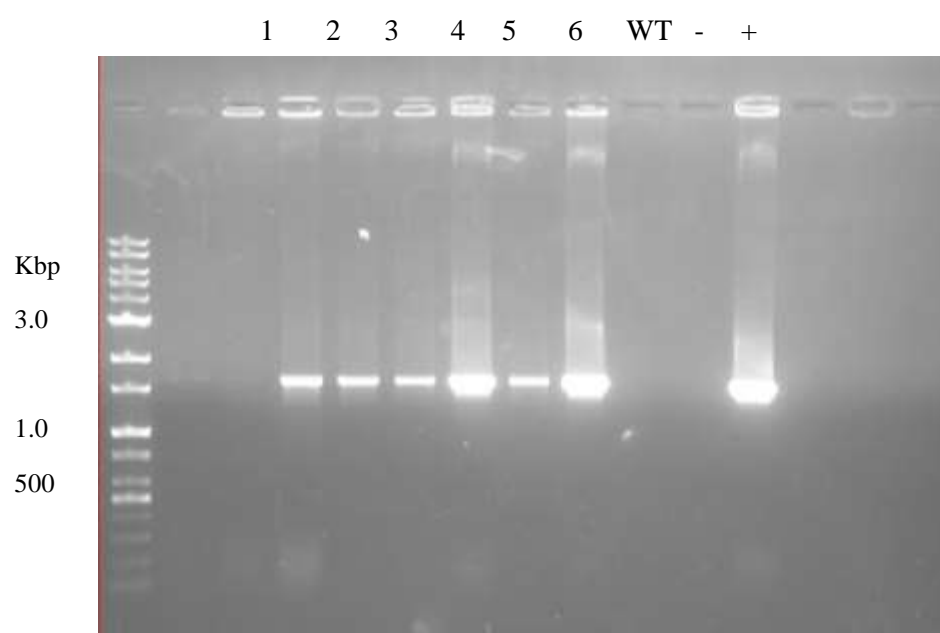

a. 3

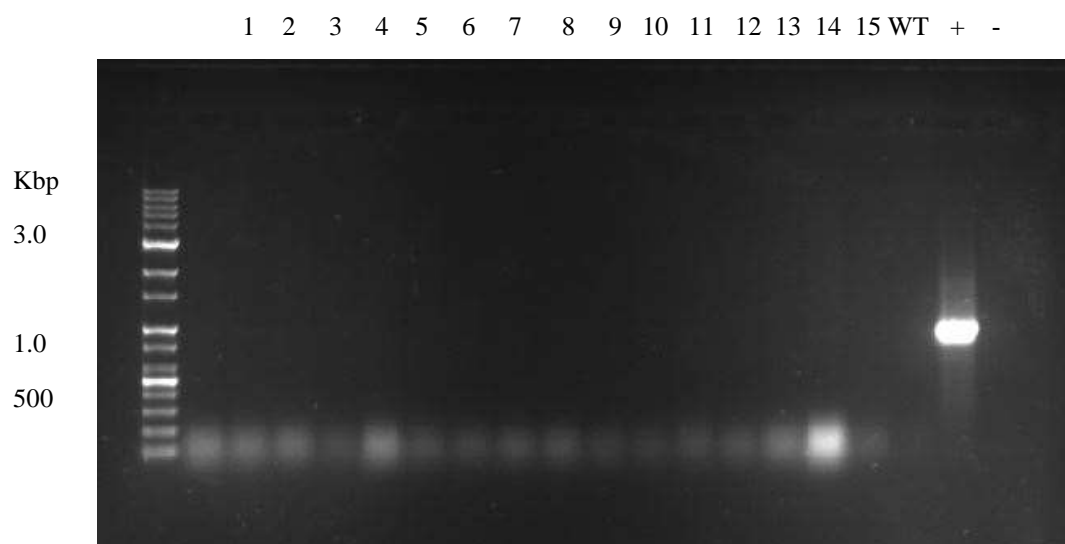

**b. 1**

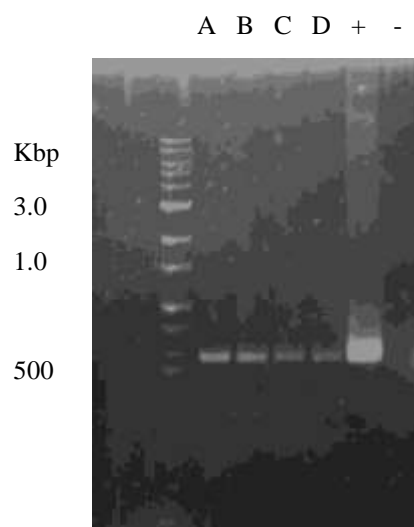

**b. 2**

**b. 3**

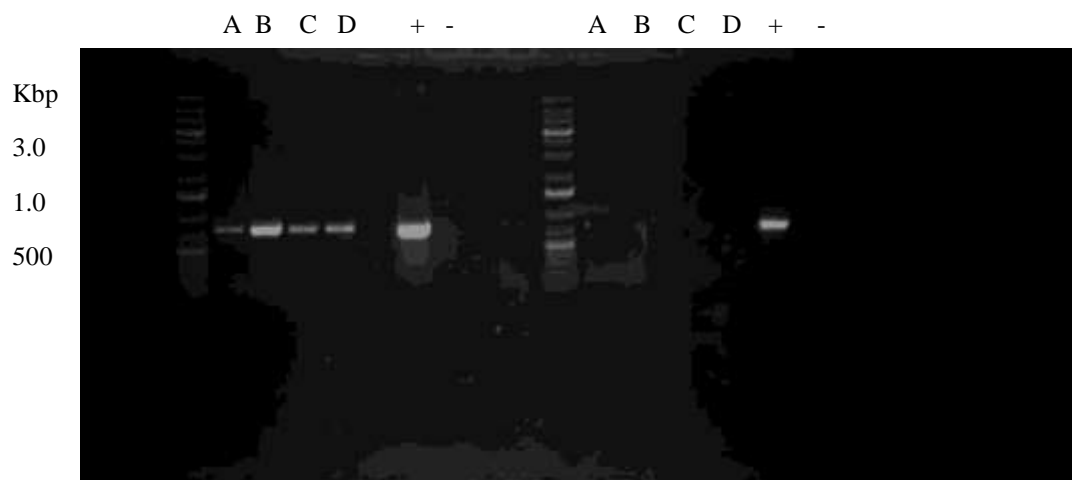

Supplement: S1 Raw images — (PDF) [file pone.0268036.s003.pdf]
